# CRYβA3/A1-Crystallin Knockout Develops Nuclear Cataract and Causes Impaired Lysosomal Cargo Clearance and Calpain Activation

**DOI:** 10.1371/journal.pone.0149027

**Published:** 2016-02-10

**Authors:** Shylaja Hegde, Robert A. Kesterson, Om P. Srivastava

**Affiliations:** 1 Department of Vision Sciences, School of Optometry, University of Alabama at Birmingham, Birmingham, Alabama, United States of America; 2 Department of Genetics, University of Alabama at Birmingham, Birmingham, Alabama, United States of America; University of Colorado Denver School of Medicine, UNITED STATES

## Abstract

βA3/A1-crystallin is an abundant structural protein of the lens that is very critical for lens function. Many different genetic mutations have been shown to associate with different types of cataracts in humans and in animal models. βA3/A1-crystallin has four Greek key-motifs that organize into two crystallin domains. It shown to bind calcium with moderate affinity and has putative calcium-binding site. Other than in the lens, βA3/A1 is also expressed in retinal astrocytes, retinal pigment epithelial (RPE) cells, and retinal ganglion cells. The function of βA3/A1-crystallin in the retinal cell types is well studied; however, a clear understanding of the function of this protein in the lens has not yet been established. In the current study, we generated the βA3/A1-crystallin knockout (KO) mouse and explored the function of βA3/A1-crystallin in lens development. Our results showed that βA3-KO mice develop congenital nuclear cataract and exhibit persistent fetal vasculature condition. At the cellular level KO lenses show defective lysosomal clearance and accumulation of nuclei, mitochondria, and autophagic cargo in the outer cortical region of the lens. In addition, the calcium level and the expression and activity of calpain-3 were increased in KO lenses. Taken together, these results suggest the lack of βA3-crystallin function in lenses, alters calcium homeostasis which in turn causes lysosomal defects and calpain activation. These defects are responsible for the development of nuclear cataract in KO lenses.

## Introduction

Crystallins (α, β and γ) are structural proteins of the ocular lens, and are required for maintaining the transparency and refractive power of the lens [1]. Initially, crystallins were considered to be present only in the lens, but are now known to be expressed in other tissues [2, 3]. The function of α-crystallins is well established, they are small heat-shock proteins with chaperone activity [4]; however, the function βγ-crystallins is not as clear. β- and γ-crystallins share a common core protein structure with two similar domains, each composed of two characteristic Greek key-motifs [5]. The β-crystallins are characterized as oligomers (the molecular mass of monomers from 22–28 kDa) with molecular weight ranging up to 200 kDa. β-crystallin family consists of three basic (βΒ1, βΒ2, βΒ3) and four acidic (βΑ3/A1, βA2, βA4) proteins [6–8]. All the β-crystallin subunits are homologous but contain different N- or C-terminus. Basic β-crystallins have both N-terminal and C-terminal extensions, whereas acidic β-crystallins have only N-terminal extensions. β-crystallin reported to bind calcium with moderate affinity. Although, it does not have well defined calcium-binding structural motifs such as EF-hand or helix-loop-helix like calmodulins and troponin C, calcium-binding have been detected in high molecular weight β-crystallin (β_H_-crystallin) [9]. The calcium-binding sequences (D/NXXS), which is conserved in other members of the βγ-superfamily from lower organisms such as protein S (from Myxococcus Xanthus, a gram negative soil bacterium) or sperulin 3a (from Physaraum polycephalon), is not conserved in vertebrate’s β-crystallins [10, 11]. However, a similar sequence (DNVRS) on first Greek motif of βΑ3/A1protein has been proposed as a calcium-binding motif [9].

βΑ3/A1-crystallin is unique among other crystallins, it is composed of 2-polypeptides that are translated from single mRNA using an alternate start site with an additional 17 amino acid residue on the N-terminus of βA3 [12]. In rodents, the expression of βΑ3/A1-crystallin is detected at E12.5 in the posterior portion of the lens vesicle. In adult lens, it expresses specifically in fiber cells and is very critical for lens development since many different mutations in the βΑ3-gene result in the development of congenital cataracts in humans and in animal models [13–18]. Even though βA3-crystallin is an important structural protein and is critical for lens development, its exact role in lens development and maintenance of lens transparency are not known. Earlier studies from our laboratory suggested that βΑ3/A1-crystallin possesses a serine-type protease and autocatalytic activities [19, 20].

Other than in the lens, βΑ3/A1 expression is also detected in retinal astrocytes, retinal pigment epithelial cells (RPE), and retinal ganglion cells [21]. The function of βΑ3-crystallin in retinal astrocytes and RPE has been thoroughly explored [22–26]. Using a βΑ3-mutant (Nuc1) rat and genetically modified mouse models, βΑ3/A1 is shown to be a lysosomal resident protein that regulates multiple cellular processes in astrocytes and RPE by modulating specific signaling pathways mediated by V-ATPase. Furthermore, βΑ3/A1-crystallin interacts with V-ATPase (a proton pump), and plays an important role in regulating V-ATPase function in maintaining the pH of the endolysosomal system [23–26].

To understand the physiological role of βΑ3-crystallin in the lens, in this study we have generated a βΑ3 knockout (KO) mouse. Here, we report the phenotypic characterization and a potential molecular mechanism of cataract development in complete βΑ3 KO mice. Our results showed that βΑ3 KO mice develop congenital nuclear cataract and persistent fetal vasculature syndrome. At the cellular level, KO lenses exhibit multiple defects such as impaired degradation of nuclei, mitochondria, and autophagic cargos, lysosmal dysfunction, altered expression and assembly of cytoskeleton proteins and, increased calcium level and increased expression and activity of calpain-3. Taken together, our results suggest that βA3-crystallin is required for clearing the cargo from the cell by regulating the cytoplasmic calcium concentration. In the absence of βA3-crystallin the cytoplasmic calcium concentration is altered and it causes i) lysosomal dysfunction and cargo accumulation, ii) calpain activation and cytoskeletal defects. These defects are likely responsible for the development of congenital nuclear cataracts in βA3-KO mice.

## Materials and Methods

### Ethics Statement

All animal experiments were performed in accordance with protocols approved by the Institutional Animal Care and Use Committee (IACUC) of the University of Alabama at Birmingham (Protocol no. 130208393). Mice were housed in pathogen-free facilities at the University of Alabama at Birmingham.

### Generation of βA3/A1-Crystallin KO Mice

A CRYβA3/A1 targeted C57BL/6N ES clone was purchased from the European Conditional Mutagenesis Program (Project ID 89328). Additional quality control data and protocols for generating this clone can be found at http://www.mousephenotype.org/martsearch_ikmc_project/martsearch/ikmc_project/89328. ES cells were injected into C57BL/6J blastocysts to generate male chimeras, which were then bred to C57BL/6J females to establish germline transmission of the “knockout first” reporter tagged Cryba1^tm1a(EUCOMM)Hmgu^ allele (aka βA3- or KO allele). (This experiment was done at the Transgenic and Genetically Engineered Model Core facility of the University of Alabama, Birmingham). Heterozygous βA3-/+ animals were intercrossed to produce homozygous mutants (βA3-/-). PCR amplification was used to detect the targeted KO allele versus WT allele.

### PCR Analysis

Genomic DNA was extracted from mouse tail biopsies using a DNA isolation kit (Qiagen). The primers used for genotyping was; Primer 1 (Forward) 5’GGCAGCTGGATTGGTTATGAACACC3’ Primer 1 (Reverse) 5’GTACTCTTGTCAACAACAGAGTAAATGG 3’. This primer is used to detect the WT copy versus KO copy of the CRYβA3/A1 gene. PCR amplification was done using Taq polymerase (Sigma-Aldrich) using a PCR cycle of 94° for 3 min, followed by 30 cycles of 94° for 15 sec, 58° for 30 sec and 72° for 45 sec, followed by 72° for 10 min.

### mRNA Expression Analyses

Lenses were isolated and briefly washed with PBS and immediately homogenized with TRIZOL reagent (Invitrogen) on ice. RNA extraction was carried out using an RNA isolation kit (GE Life Sciences) following the manufacturer’s protocol. One microgram of total RNA was reverse transcribed using a cDNA synthesis kit (Invitrogen). cDNA was amplified using random primers. In RT-PCR, βA3-gene-specific primer 2 (Forward) 5’TGAGCGTCTCATGTCTTCC 3’ Primer 2 (Reverse) CCACTGGCGTCCAATAA3’was used to detect the mRNA product. β-actin was used as an internal control.

### Immunoblotting

Isolated lenses (n = 6, from wild type, heterozygous and KO mice) were weighed and homogenized in a radioimmunoassay buffer (RIPA) [Sigma-Aldrich] containing protease inhibitor cocktail tablets (Roche). Protein concentration was determined using a BCA protein assay kit (Pierce Biotechnology). 50–100 ug of protein was electrophoretically separated using 12% SDS-PAGE and transferred to a PVDF membrane (BioRad). The blots were blocked with 5% of BSA in PBS for 1 hr at room temperature, followed by overnight incubation with the primary antibody at 4°C. The primary antibodies used in this study were: anti-βA3 crystallin (1:3000) (Santacruz biotechnology); anti-P62/SQSTM (1:500) and anti-ubiquitin (1:500) (Sigma-Aldrich); and LC3 (1:500), β-actin (1:1000), and tubulin (1:1000), all from Cell Signaling Technology. After three washes with PBST (PBS+0.1% Tween 20), blots were incubated with a secondary antibody, either an HRP-conjugated (1:1000) (Cell Signaling Technology), or an IR dye-conjugated antibody (1:10,000) from LI-COR. After three washes, specific protein bands were visualized on a Li-Cor Odyssey system (in the case of the HRP-conjugated secondary antibody, a chemiluminecent substrate from Li-Cor was used).

### Slit Lamp Microscopy

Animal’s pupils were dilated with eye drops containing 1% tropicamide and 5% Phenylphrine hypochloride. After 15–20 minutes, eyes were examined using a micron IV slit lamp (Phoenix Research labs, California) at the Vision Science Research Core Facility of the University of Alabama at Birmingham.

### Cell Culture

Mouse lens epithelial cells were cultured as previously described [27]. Briefly, lenses were extracted from 4–5 weeks old mice, (except for co-localization study 4–5 months old lenses were used) rinsed in PBS, placed in 0.25% trypsin, and incubated at 37°C for 3 hrs. The cells were recovered by centrifuging at 2000 rpm, washed with M199 medium, and cultured in 30 mm plates with M199 medium (Invitrogen) containing 10% fetal bovine serum (Hyclone). Cultures were fed three times per week. For immunofluorescence studies, the cells were plated on 8-well Lab-Tek-II chambered slides (ThermoScientific), and next day the cells were washed with PBS and fixed with 4% formaldehyde in PBS.

### LECs Immunofluorescence

The fixed cells were washed with PBS and blocked with 3% BSA containing 0.3% Triton-X-100 for 30 min. To visualize the distribution of LC3-II and p62/SQSTM, the cells were incubated overnight with polyclonal anti-LC3-II (1:200) and anti-p62/SQSTM (1:200) antibodies. To visualize cytoskeletal integrity, polyclonal anti-tubulin (1:500) and anti-alpha spectrin (1:500) antibodies were used. To detect the calpain lavel anti-calpain LP-85 (1:200) [Sigma-Aldrich] was used. For lysosome and autophagosomal co-localization Lamp-II and AlexaFluor 488 labeled LC3B/MAP1LC3B (1:250) (Novus Biologicals) were used. An AlexaFluor 488-conjugated or AlexaFluor 590- conjugated anti-rabbit IgG (Invitrogen) was used as the secondary antibody. To visualize the distribution of F-actin, cells were incubated with Texas Red-Phalloidin (1:50) (Molecular Probes, Invitrogen) by following the manufacturer’s instructions. The cells were mounted with aqueous mounting media (Southern Biotech) and examined with a Nikon confocal microscope, or, in some cases, with a Zeiss Axioplan 2 fluorescence microscope equipped with a CCD camera.

### Immunohistochemistry

For immunohistochemical studies, mouse eyes (n = 4) were fixed in 4% paraformaldehyde for 24 hrs, rinsed in PBS, and were paraffin-embedded. 4-μm thick sections were prepared and then de-paraffnized and rehydrated using PBS, followed by heat-induced epitope retrieval in 10 mM sodium citrate and 0.2% Tween 20. The sections were rinsed with water and blocked in 3% (w/v) BSA in PBS for 30 min, and incubated overnight with the desired primary polyclonal antibodies. The sections were washed with PBS and incubated with AlexaFluor 488-conjugated anti-rabbit secondary antibodies (Invitrogen) at 1:1000 dilutions in PBS for 1 hr at room temperature. The sections were then washed in PBS and stained with Hoechst 33342 nuclear stain (1:100 dilutions) for 1 min and rinsed with PBS. For the negative controls, the individual primary antibodies were omitted. The sections were mounted with aqueous mounting media and examined with a Nikon confocal microscope, or in some cases, with a Zeiss Axioplan 2 fluorescence microscope equipped with a CCD camera.

### Electron Microscopy

Lenses were fixed in 100 mM phosphate-buffered saline pH 7.4 containing 4% paraformaldehyde and 0.05% glutaraldehyde (Electron Microscopy Sciences) for 2 hrs at room temperature and then overnight at 4°C. The fixed lenses were washed with Millipore water and dehydrated by ascending ethanol gradients followed by infiltration with absolute ethanol: (London Resin [LR] white 1:1) overnight at 4°C. The lenses were removed and transferred to gelatin capsules containing fresh LR white and were allowed to polymerize for 24 hrs at 45–50°C. Ultra-thin (70 nM) LR white-lens sections were collected on nickel mesh grids, and the sections were imaged on an FEI 120 kv Spirit TEM (FEI-Hillsboro, WA) and collected using an AMT (AMT-Woburn, MA) digital camera.

### Calcium imaging

Lenses were extracted from 4–5 months old WT and KO mice and lens epithelial cells were cultured as described above. Cells were incubated with Calcium orange (4 μM, Molecular Probes; C3015) for 45 minutes, to allow the Calcium dye to enter intracellular organelles. The dye was removed and cells were washed once with PBS, and immediately fixed with 4% paraformaldehyde and imaged with a Zeiss Axioplan 2 fluorescence microscope equipped with a CCD camera.

### Mass Spectrometry

Desired SDS gel bands were excised and trypsin digested at 12.5 ng/μl concentration of the enzyme (Promega Gold Mass Spectrometry Grade). Tryptic peptides were extracted from the gel pieces using a 1:1 mixture of 5% formic acid and 50% aqueous acetonitrile twice for 15 min. Extracts were pooled and evaporated to dryness. The samples were then resuspended in 20 μl of 0.1% formic acid prior to mass spectrometric analysis.

An aliquot (5 μL) of each digest was loaded onto a Nano cHiPLC 200μm x 0.5mm ChromXP C18-CL 3μm 120Å reverse-phase trap cartridge (Eksigent, Dublin, CA) at 2 uL/min using an Eksigent autosampler. After washing the cartridge for 4 min with 0.1% formic acid in ddH_2_0, the bound peptides were flushed onto a Nano cHiPLC column 200μm x 15cm ChromXP C18-CL 3μm 120Å (Eksigent, Dublin,CA) with a 45 min linear (5–50%) acetonitrile gradient in 0.1% formic acid at 1000 nl/min using an Eksigent Nano1D+ LC. (Dublin, CA). The column was washed with 90% acetonitrile-0.1% formic acid for 10 min and then re-equilibrated with 5% acetonitrile-0.1% formic acid for 10 min. The AB SCIEX 5600 Triple-Tof mass spectrometer (AB-Sciex, Toronto, Canada) was used to analyze the protein digest. The IonSpray voltage was 2300 V and the de-clustering potential was 80 V. Ionspray and curtain gases were set at 10 psi and 25 psi, respectively. The interface heater temperature was 120°C. Eluted peptides were subjected to a time-of-flight survey scan from 400–1250 m/z to determine the top twenty most intense ions for MS/MS analysis. Product ion time-of-flight scans at 50 msec were carried out to obtain the tandem mass spectra of the selected parent ions over the range from m/z 400–1500. Spectra were centroided and de-isotoped by Analyst software, version TF (Applied Biosystems). In-house MASCOT database searches were carried out against the mouse genome on the NCBInr database. The mass tolerances for precursor scans and MS/MS scans were set at 0.05 Daltons. One missed cleavage for trypsin was allowed. A fixed modification of carbamidomethylation was set for cysteine residues, and a variable medication of oxidation was allowed for methionine residues. Proteins with at least one individual peptide MOWSE score of <40 were considered significant.

### Calpain Assay

Lenses were homogenized in 10 mM HEPES (pH 7.2), 10 mM DTT, 1 mM EDTA, and 1 mM EGTA. The homogenates were centrifuged at 14,000 rpm for 15 min, and cytoplasmic fraction was used to determine calpain activity per the manufacturer’s Calpain-Glo-Protease assay protocol (Promega). The luminescence from the substrate cleavage was plotted as a Relative Light Unit /mg of protein.

## Results

### Generation and Characterization of CRYβA3/A1 Knockout Mice

**Fig 1:**
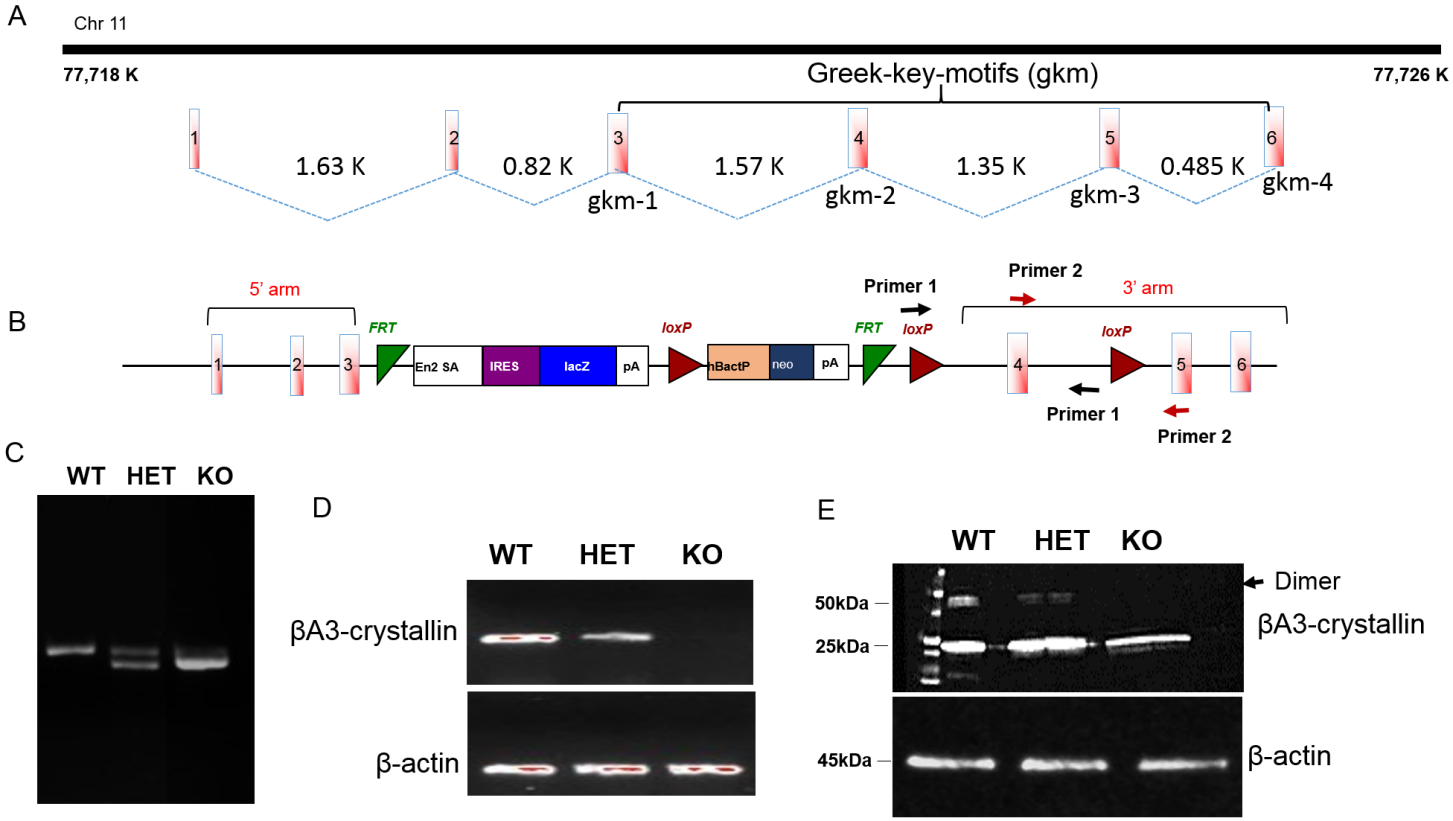
Generation of CRYβA3/A1 knockout mice. **A**. Organization of the CRYβA3 gene on chromosome 11, consisting of 6 exons flanked by introns. Exon 3–6 are the critical exon that encode four Greek key-motifs I-IV. **B**. Schematic of the first knockout strategy used for generating βA3/A-crystallin KO mice. Shown is the Cryba1^tm1a(EUCOMM)Hmgu^ “knockout first” allele with a (FRT)-flanked selection cassette inserted into the third intron of the βA3/A1 gene, which traps the transcript through the Engrailed-2-splice acceptor (En2 sA) and truncates it through the SV40 polyadenylation signal. Primer specific to the neomycin resistance gene was used to confirm the disruption of the βA3/A1 gene. **C**. Genotyping result for WT, HET and HOM KO mice. **D**. Confirmation of the deletion of βA3/A1 mRNA in KO lenses by qRT-PCR. **E**. Western blot analysis using a polyclonal βA3/A1-crystallin antibody. Lack of band at 50 kDa dimer position (βA3-crystallin normally exist as a dimer) in KO lenses and reduced intensity in HET lenses.

To explore the possible physiological function of βA3/A1 crystallin in lens development, we generated a CRYβA3/A1 KO mouse model using a targeted ES cell clone from the European Conditional Mouse Mutagenesis Program (EUCOMM). Fig 1A, shows the arrangement of CRY βA3/A1 gene on the chromosome (Chr 11). CRY βA3/A1 gene consists of six exons spanning ~7 kilobases. The KO mouse was constructed based on the “knockout first strategy” of gene targeting (Fig 1B) [28]. The strategy introduced a Flip-recombinase target (FRT)-flanked selection cassette into the third intron of the CRY βA3/A1 gene in order to inhibit transcription of the full length gene (mediated through aberrant splicing to the Engrailed-2 splice acceptor (En2 sA) element and premature termination through downstream SV40 polyadenylation (pA) signals). Genotyping confirmed the disruption of the CRYβA3/A1 gene and to distinguish the WT, HET (heterozygous) and KO mice (Fig 1C). To verify that no βA3-crystallin is produced by the Cryba1^tm1a(EUCOMM)Hmgu^ mutant allele, lenses from 4-weeks old WT, HET and KO mouse lenses were analyzed by RT-PCR and Western blotting. In RT-PCR, CRYβA3/A1 gene-specific primer failed to amplify the βA3 gene product in KO lenses, and in HET lenses the amount of amplified product was reduced by 50% compared to the amount of amplification product in WT lenses (Fig 1D). In western blot analysis, the anti-βA3-crystallin antibody reactivity corresponding to a dimer (50 kDa) was completely absent in KO and was reduced to 50% in HET compared to WT (Fig 1D). However, at the 25 kDa position, eventhough the intensity of band was drastically reduced in KO lenses, immunoreactivity was still observed at that position similar to that in WT and HET lenses. To rule out the possibility that the signal at 25 kDa was not due to a non-specific reactivity of the antibody, experiment was repeated with a monoclonal βA3-crystallin antibody, however similar results were observed. This suggests that a small amount of βA3-crystallin protein is still being produced in KO lenses.

### CRYβA3/A1-Deficient Mice Develop Congenital Nuclear Cataracts and Show Persistent Fetal Vasculature

**Fig 2:**
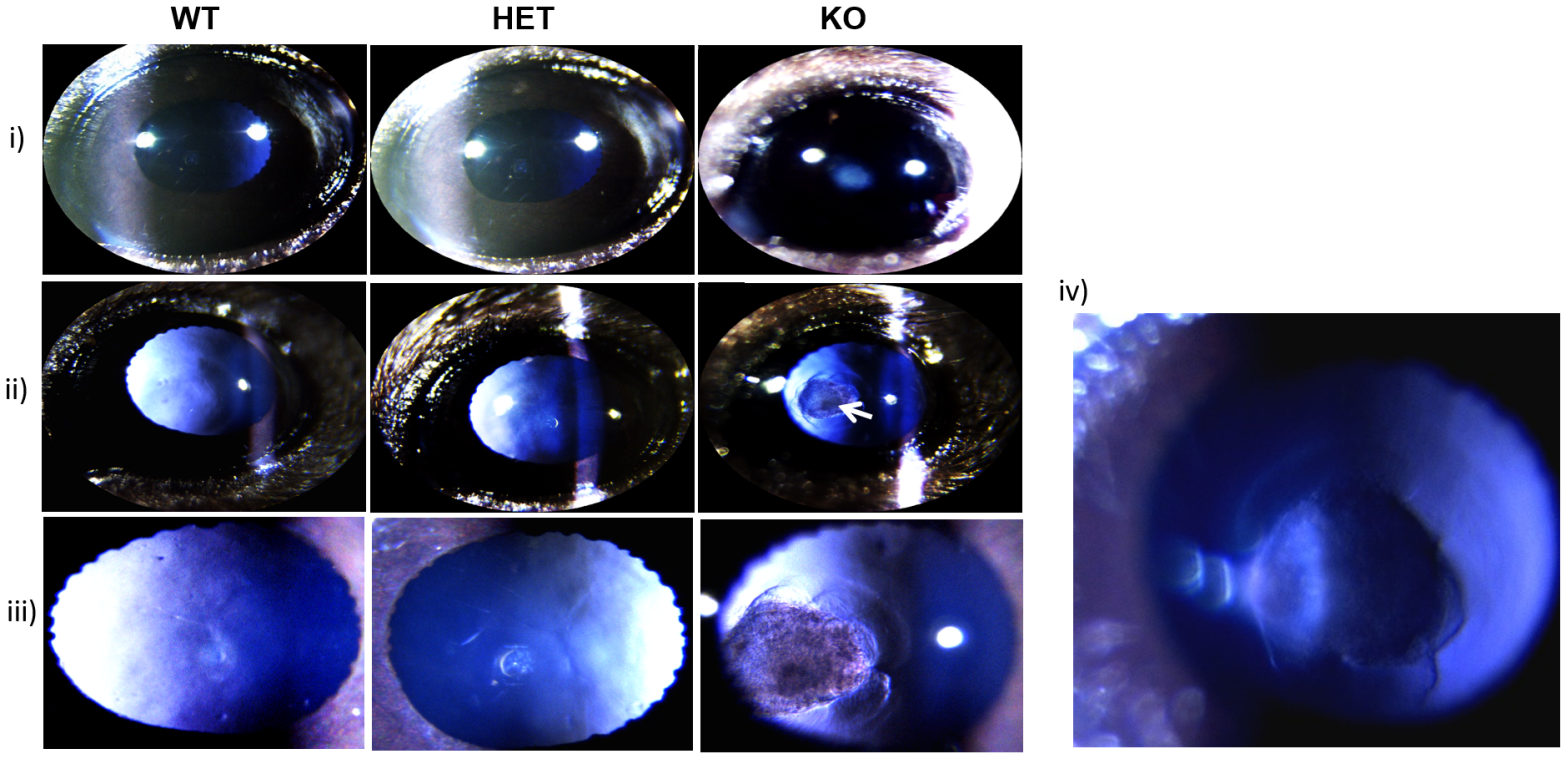
Slit-lamp examination of lenses of WT, HET and KO. 4-weeks-old mice were examined with a Micron IV-slit lamp after dilation of the pupils. **i)**. Slit-lamp images of the WT, HET and KO mice eyes from the front view. Clear nuclear cataracts were observed in the center (nuclear region) of the KO lenses (n = 3 animals). **ii)**. Images of the lenses with the slit-beam incident on the side of the eye. Images showing the presence of a fibrous mass (shown by an arrow), fetal hyaloid artery retention (condition called Persistent Fetal Vasculature) posterior to the lens. **iii)**. The Y-shaped suture line is distorted in KO lenses compared to a distinct Y-shaped suture line in WT and HET lenses. **iv)**. Illumination from the back of the lens showing the attachment of the hyaloid artery in a KO lens. (n = 3 animals).

At 4-weeks of age, the external morphology of the whole eye and dissected lenses appeared to be normal in KO mice. However, eye balls from KO were 20–30% smaller in size than those of HET and WT mice. Lenses from these eyes also weighed 25–30% less than those of WT lenses. Initial analysis also showed that the orientation of the lens fibers was altered in KO lenses compared to orientation of lens fibers in WT or HET lenses (S1 Fig). Next, eyes of KO, HET and WT mice at 2–4 weeks were examined by Slit-lamp, using Micron IV microscope. The nuclear cataract was apparent in KO lenses (Fig 2A panel (i)). In fact cataract was observed in 100% of the KO pups (n = 20) as soon as they opened their eyes, even without the aid of the instrument suggesting that KO mice develop congenital nuclear cataract. Surprisingly, the slit-lamp analysis of KO lenses indicated the presence of a fibrous mass posterior to the lens [Fig 2A panel (ii-iv)], which was identified as the retention of the hyaloid artery, an artery in the fetal state that nourishes the developing lens, extending from the optic disc to the center of the posterior of the lens. This artery usually regresses before birth. The condition in which the hyaloid artery is retained is known as persistent fetal vasculature (PVF). The p62 staining of 1-month old lenses further confirmed the PVF in KO lenses (data not shown). In addition, the distinct Y-suture line observed in WT and HET lenses was distorted in KO lenses (Fig 2A panel (iii)).

### Nuclear DNA Degradation Process is Impaired in KO Lenses

**Fig 3:**
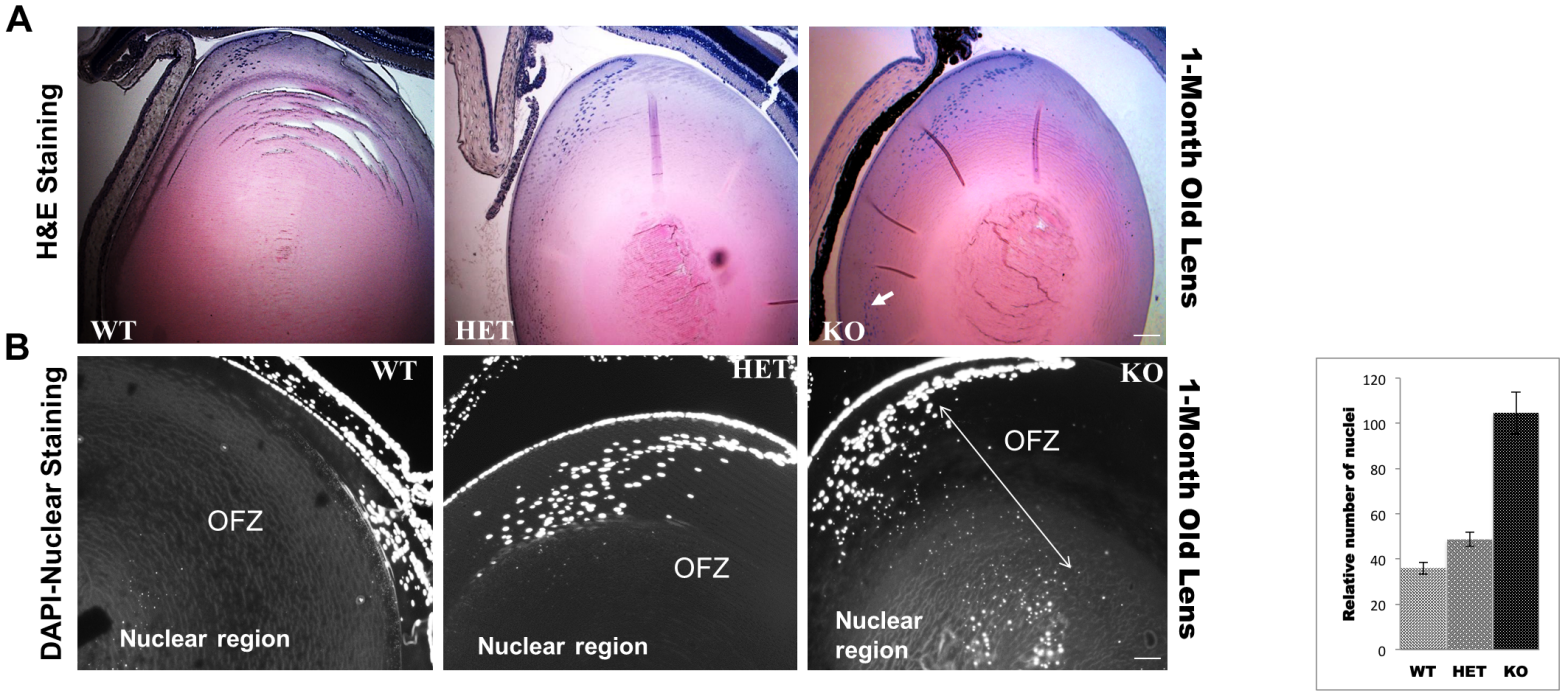
Nuclear degradation process is impaired in CRYβA3/A1KO mice. **A**. H&E staining of 1-month old WT, HET and KO mice lens sections. An arrow in KO lens image show an abnormally high number of nuclei compared to WT and HET lenses. **B**. DAPI-staining of 1-month old lens sections of WT, HET and KO mice. DAPI-staining shows an accumulation of nuclei and nuclear debris in the OFZ (organelle free zone) and in nuclear region of KO lenses (indicated by an arrow). The bar graph on the right shows the quantification of nuclei. Data represent mean ± S.D in n = 4 independent experiments. **, P<0.01 by student’s t test. Scale bars: A: 100 μm, B: 50 μm.

Comparison of H&E staining of 4-weeks old KO mice lenses with age-matched HET and WT lenses showed a major disruption in the center of the KO lenses. In addition, KO lenses showed the presence of an abnormally high number of nuclei in the anterior side (shown by an arrow) of lens (Fig 3A). To further confirm this observation, the 4-weeks old lens sections were stained with the DAPI-nuclear stain. In KO lenses, the primary fiber cells in the center and the secondary fiber cells in the outer most layers of the OFZ retained their nuclei. Although no intact nuclei were observed in the OFZ, accumulated degraded DNA debris was evident (Fig 3B). Quantification of nuclei indicated that almost 2–3 folds more nuclei were retained in KO lenses compared to the control lenses (Fig 3B, right panel). This suggests that in the absence of βA3/A1-crystallin, the programmed nuclear degradation process is dysregulated.

### Autophagic Cargo Accumulates in CRYβA3/A1 KO Lenses

**Fig 4:**
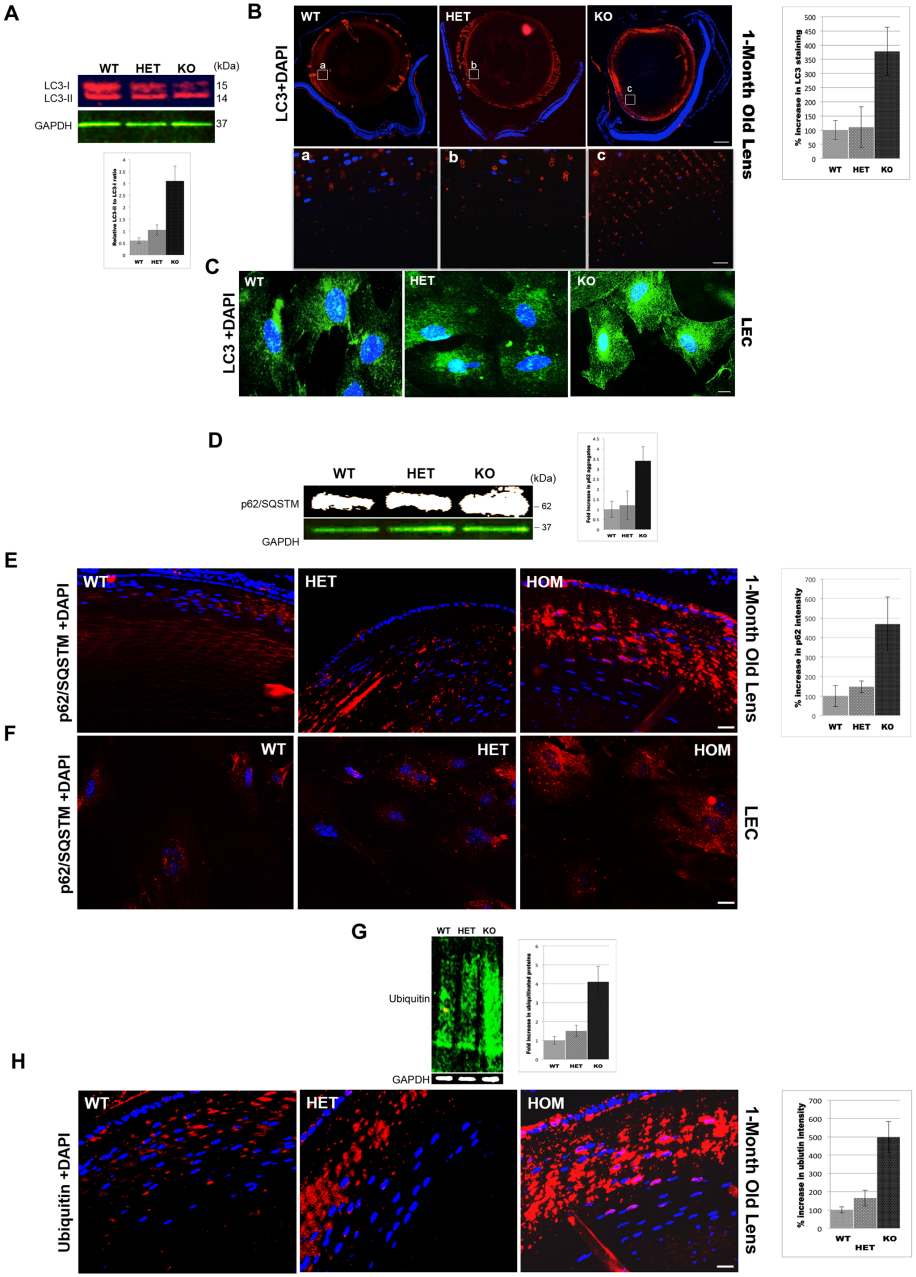
Autophagic Markers Accumulate in CRYβA3/A1 KO mouse. Detection of LC3 levels in KO mice lenses. **A**. Western blot of 1-months old lens homogenates probed with LC3-antibody (red) and GAPDH (green). Compared to WT and HET lenses, the ratio of LC3-II to LC3-I was greater in KO lenses. The bar graph below is the quantification of relative ratio of LC3-II to LC3-I. Data represent mean ± S.D in n = 3 animals. *, P<0.05 by student’s t test. **B**. Immunohistochemical staining of 1-month-old lens sections with the LC3 antibody. (LC3: red, DAPI nuclear stain: blue) Intensity of LC3 staining was increased, 2–3 folds in KO lenses compared to WT and HET lenses. The OFZ of KO lenses (panel c) showed positive LC3 staining compared to the corresponding region in WT (panel a) and HET (panel b) lenses. Bar graph on the right panel represent quantification of LC3 staining (mean ± S.D) in n = 4 lenses. *, P<0.05 by student’s t test. **C**. Cultured Lens Epithelial Cells (LECs) from WT, HET and homozygous KO mice were examined by immunofluorescence using an anti-LC3 antibody (green). **D**. Analysis of p62/SQSTM expression in KO mice lenses. Western blot of 1-months lens homogenates probed with the p62-antibody (white) and GAPDH antibody (green). The bar graph on the right shows the quantification of p62 intensity. Data represent mean ± S.D in n = 3 animals. *, P<0.05 by student’s t test. **E**. Immunohistochemical analysis of p62 distribution in mouse lens sections. LEC and lens fiber cells of the KO show increased p62 expression (red) and larger p62 aggregates compared to the LEC and lens fiber cells of WT and HET lens. The bar graph on the right shows the quantification of p62 intensity. Data represent mean ± S.D in n = 4 lenses. *, P<0.05 by student’s t test. **F**. Immunofluorescence analysis of p62 in LEC. KO cells show increased expression of p62 and larger sized p62 aggregates compared to HET and WT cells. n = 2, independent experiments. **G**. Western blot of lens homogenates probed with the ubiquitin antibody (green) and GAPDH antibody (white). The bar graph on the right shows the quantification of ubiquitin signal. Data represent mean ± S.D in n = 3 animals. *, P<0.05 by student’s t test. **H**. Immunohistochemical analysis of ubiquitinated proteins in mouse lens sections. Outer cortical region of KO showing drastic increase in ubiquitinated proteins (red) compared to control lenses. Bar graph on the right side is a quantification of ubiquitin signal represented as mean ± S.D in n = 3 lenses. *, P<0.05 by student’s t test. Scale bars: B: 100 um, a,b and c, E, H: 50 μm, and C, F:20 μm.

Autophagy is an important intracellular quality control process. It has been shown to be constitutively active in mouse lenses [29, 30]. Defects in autophagy lead to the accumulation of protein aggregates and organelles and have been shown to cause both age–related and congenital cataracts [31, 32]. Moreover, autophagosomes are shown to accumulate in the RPE-specific βA3-crystallin conditional knockout (cKO) mice [25]. Therefore, we next investigated the integrity of the macroautophagy pathway in KO lenses by analyzing the lipid modification of LC3 leading to membrane-associated LC3-II (15–16kDa), which is a typical hallmark of autophagosome formation. LC3-II is rapidly degraded by lysosomal proteases once the autophagosome is fused with lysosome [33]. The immunoblot of 1-month old lens homogenates using the anti-LC3 antibody showed that the ratio of LC3-II to LC3-I was higher in KO lenses than LC3-II to LC3-I ratio in WT or HET lenses (Fig 4A). The imuunostaining results also showed that LC3 was substantially increased in lens epithelial and outer cortical fiber cells in KO lens compared to corresponding region in WT or HET lenses. Moreover, in the KO lenses intense punctate staining of LC3 was observed in the inner cortical region which was absent in WT and HET lenses (Fig 4B enlarged area a, b and c). Quantification of LC3 staining demonstrated 3–4 folds increase in LC3 in KO lenses compared to WT or HET lenses (Fig 4B, left panel). Similarly, when the primary cultured LECs were immunostained with LC3 antibody, LECs derived from WT and HET lenses showed diffused cytoplasmic expression of LC3-II. In contrast, LECs from KO lenses, showed a typical morphology of cells undergoing autophagy, i.e. cells showing elevated LC3-II staining, localizing around the nucleus (Fig 4C). Since LC3-II is autophagosome-bound and is rapidly degraded as soon as it is fused to lysosomes, the accumulation of LC3-II and autophagosomes in KO lenses must be due to an induction of autophagy or a blockage in further processing of autophagosomes.

To further investigate autophagic process in KO lenses, the p62/SQSTM expression was analyzed. p62 or sequestosome 1 (p62/SQSTM1), is an LC3 interacting protein [34] and is specifically degraded during autophagy and accumulates under autophagy-deficient conditions [35, 36]. Immunoblot using an anti-p62 antibody showed a significant increase in the intensity of a 62 kDa band in KO lenses compared to WT and HET control lenses (Fig 4D). Next, immunostaining of 4-weeks old lens sections with p62-antibody showed faint p62 immunostaining in epithelial and outer cortical fiber cells of WT or HET lenses in contrast in KO lenses, the intensity of p62 in the epithelium and outer cortical fiber cells was increased tremendously (Fig 4E). Quantification of p62 immunostaining showed 4–5 folds increase in p62 accumulation compared to control lenses (Fig 4E, left panel). Consistent with this, the staining of p62 in LECs from KO lenses also increased compared to the p62 staining in LECs from WT and HET controls. In addition, the sizes of p62 speckles were much bigger in KO LECs compared to the p62 speckle size in WT and HET LECs (Fig 4F). Further, the lens homogenates probed with anti-ubiquitin antibody also showed increased accumulation of poly ubiquitinated proteins in KO lenses compared to control (Fig 4G) In addition, immunohistochemical analysis of 1-month old lens sections also exhibited a drastic accumulation of 5-fold increase in poly-ubiquitinated proteins in KO lenses compared to controls (Fig 4H). Together these results confirm that autophagic cargo accumulates in KO lenses.

### Mitochondria Accumulate in CRYβA3/A1KO Lenses

**Fig 5:**
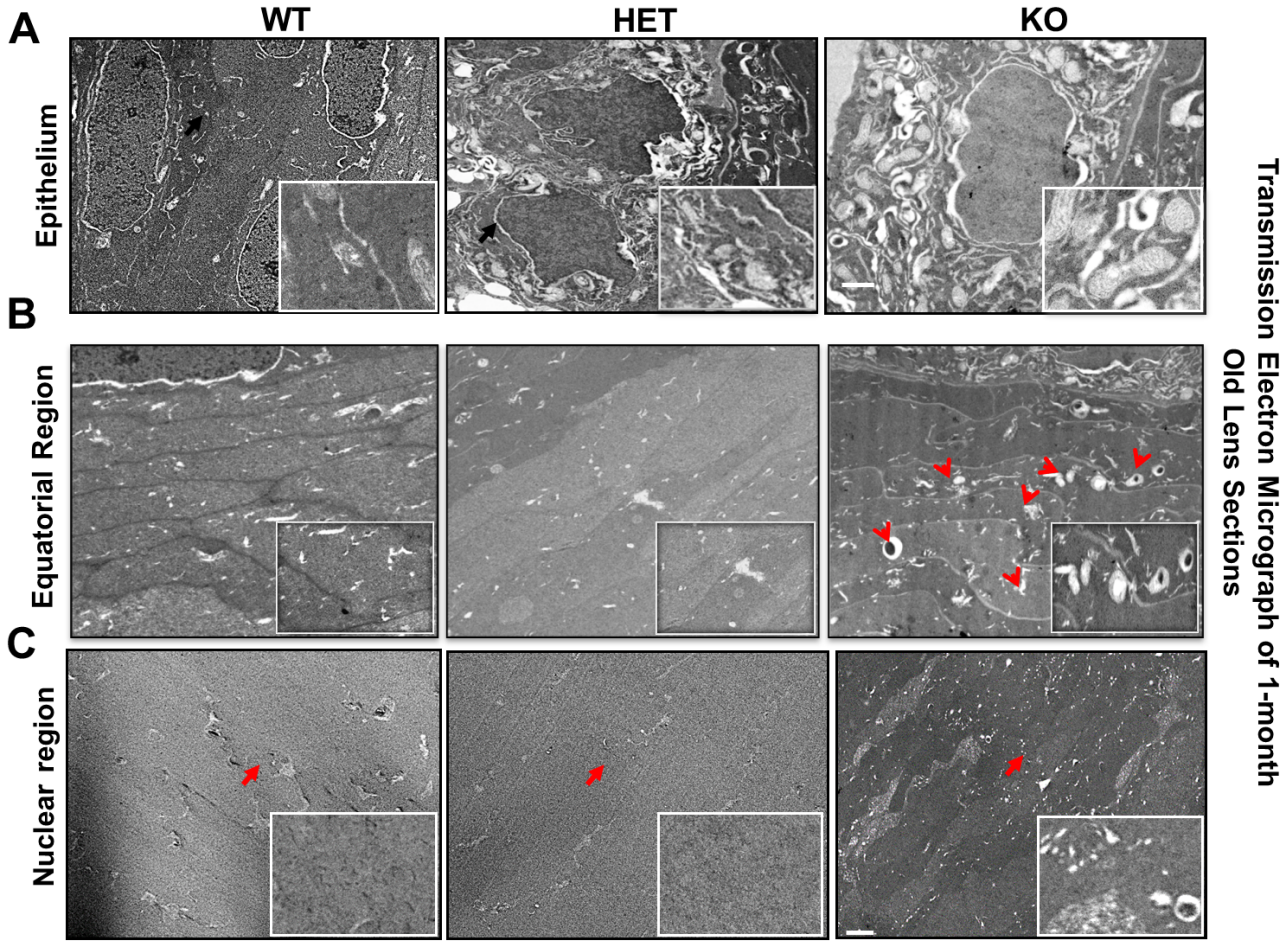
Mitochondria accumulate in CRYβA3/A1KO mice. Transmission Electronmicrographs 1-months old WT, HET and KO lenses. **A**. EM images of lens epithelium. The epithelium of KO lenses shows a number of double-membraned vesicles containing undegraded organelles, mostly mitochondria (shown by a black arrow, and also in the enlarged inset image). No such accumulation of mitochondria in the epithelium of WT or HET lenses was seen. **B**. EM images of the equatorial region of the lens. The fiber cells in KO lens show presence of mitochondria and other organelles as shown by red arrows and in enlarged inset image. **C**. EM images of the nuclear region (center) of the lens. The nuclear regions of WT and HET lenses were devoid of any cytoplasmic debris (indicated by arrows and in enlarged inset image). Cytoplasmic debris were present in the center of the KO lens (indicated by an arrow and enlarged inset image). n = 2 independent experiments. Scale bars: 2 μm and inset images 500 nm.

To obtain further insight regarding the impact of βA3 deletion on the autophagic process, an electron microscopic technique was employed. Compared to HET and WT, the electron micrographs of KO lenses showed the presence of autophagosomes (double membrane vesicles) in the cytoplasm as well as the accumulation of residual bodies containing highly indigestible organelles, mostly consisting of partially degraded mitochondria, as shown by an arrow (Fig 5A upper panel and inset images). Moreover, in KO lenses the equatorial region showed the presence of mitochondria and other organelles (Fig 5A middle panel and insets images). To further confirm that mitochondria were retained in KO lenses, the lens sections were stained with cox-iv antibody (S2 Fig). The results clearly showed the presence of positive cox-iv immunostaining in equatorial region of KO lenses compared to WT or HET lenses. Furthermore, in the KO lenses the cytoplasmic debris were detected in the center (nuclear region) of the lens compared to the nuclear region of the HET and WT lenses, which were devoid of any such debris (Fig 5C lower panel and insets images).

Together, these results further support that the cargo clearance process is impaired in KO lenses.

### Fusion of Autophagosomes with Lysosomes is normal in KO Lenses

**Fig 6:**
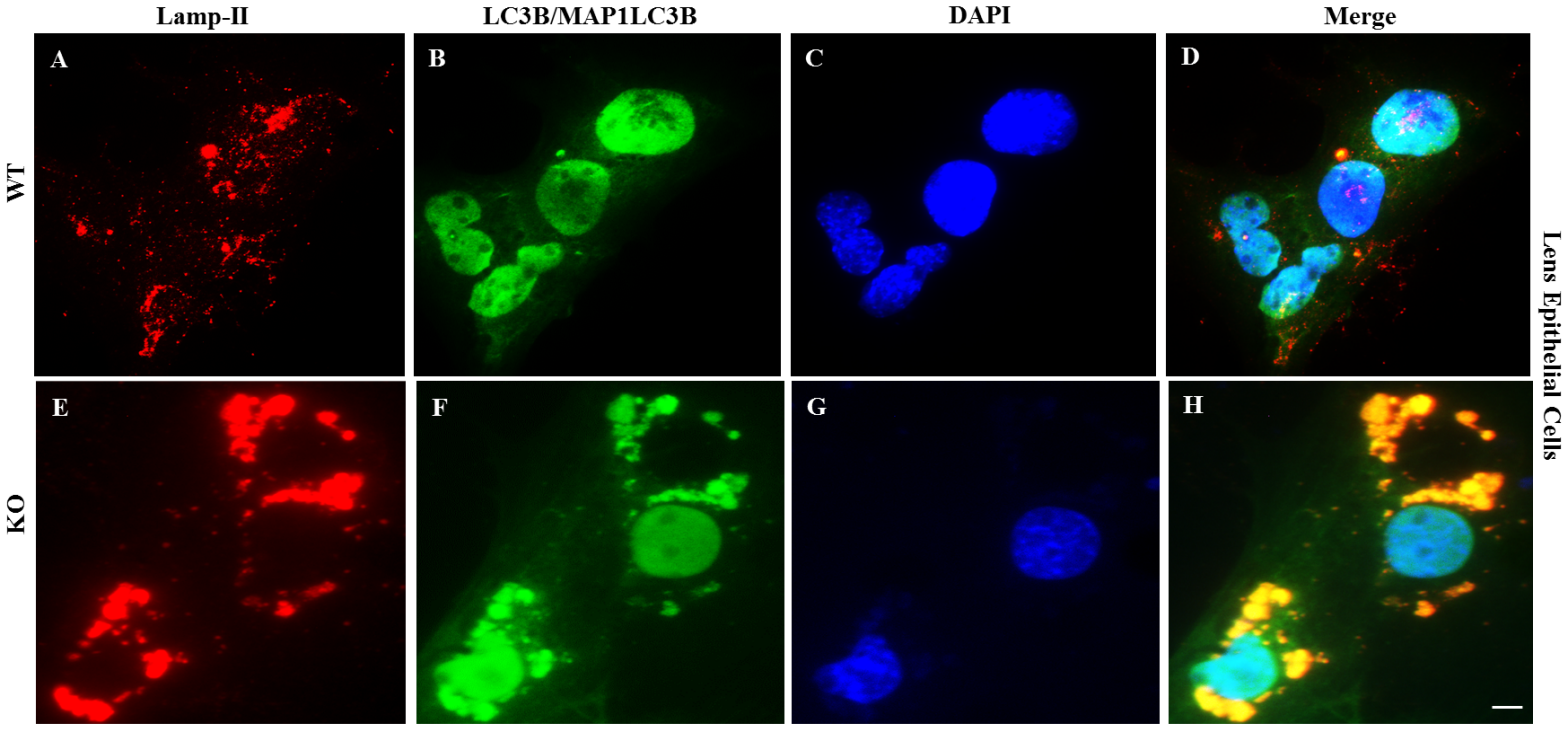
Visulaization of colocalization of lysosomes with autophagosomes. Lens epithelial cells from WT and KO lenses were cultured and immunostained with anti-Lamp-II antibody (red) to visualize lysosomes followed by staining with LC3B/MAP1LC3B antibodyconjugated with AlexaFluor 488 (green) and DAPI nuclear stain (blue) to label autophagosomes and nuclei respectively. Panels (A-D) show immunostaining of WT lens epithelial cells and (E-H) of epithelial cells from KO mice lenses. (Data is representative of 8–10 cells for each type). Scale bars: 20 μm.

Clearance of autophagosomes occurs via fusion of autophagosomes with lysosomes. We determined whether the defective clearance of autophagic substrates in KO lenses is caused by impaired autophagosome-lysosome fusion. Cultured LECs from WT and KO lenses were co-stained with the Lamp-II antibody (label lysosomes) followed by staining with the AlexaFluor 488-tagged LC3B/MAP1LC3B antibody (stain autophagosomes). Microscopic analysis revealed (Fig 6 Panel D) that the large number of autophagosomes accumulates in KO LECs and the majority of those autophagosomes were co-localized with the lysosomes (Fig 6 Panel H) thus suggesting that the fusion of auopghagosomes with lysosomes in KO lenses is normal like WT cells. However, the sizes of lysosomes in KO cells were increased relative to WT, suggesting that undigested material accumulates in the lysosomes of KO cells. Overall this result suggests that the block in autophagy in KO lenses is due to defects in lysosomal degradation process.

### Calcium Homeostasis is affected in CRYβA3/A1 KO Lenses

**Fig 7:**
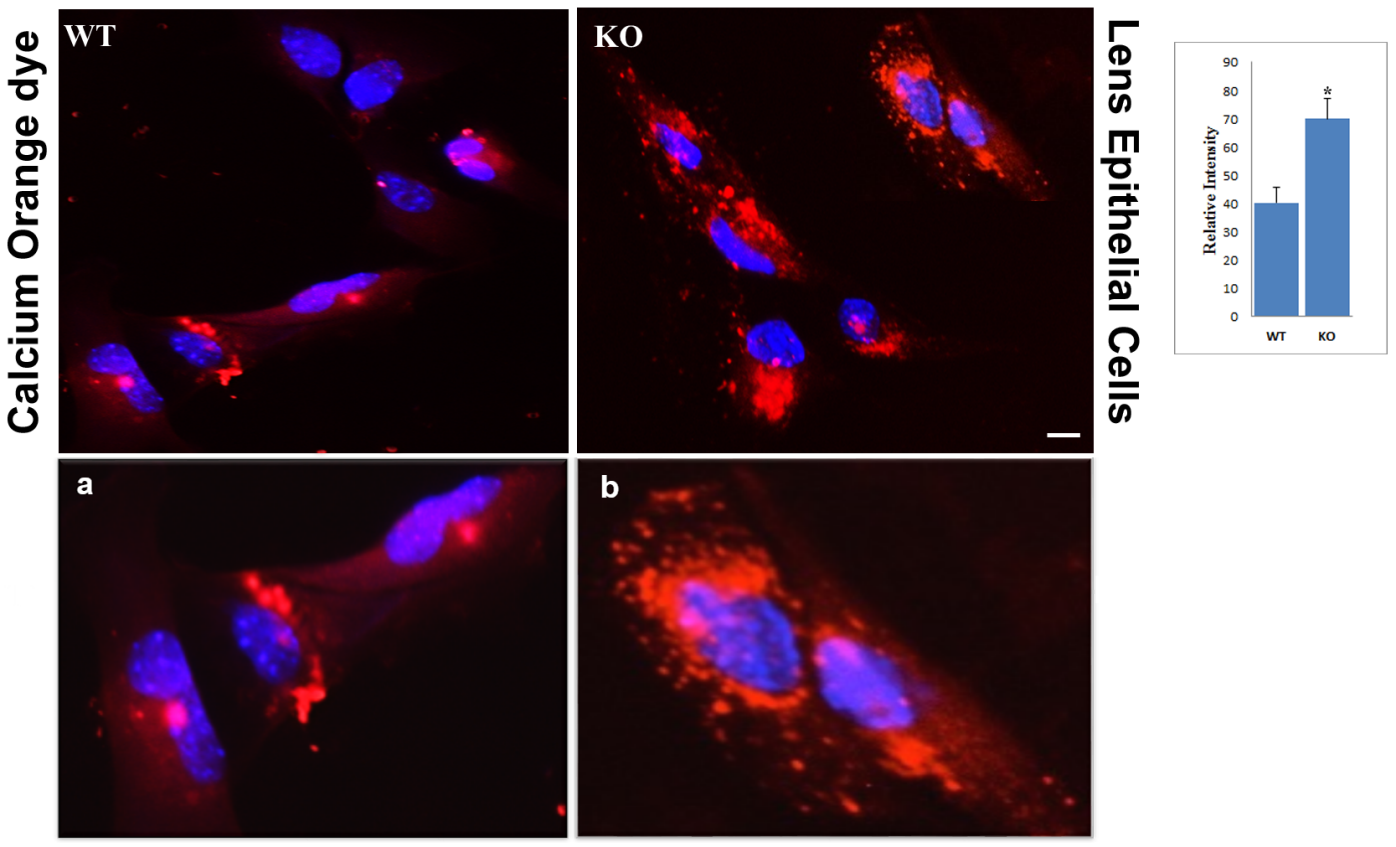
Ca^2+^ level is altered in KO cells. Cultured LECs from WT, and KO lenses were incubated with a Ca^2+^- dye, Calcium Orange (4 μM; red) for 45 minutes, during which dye enters intracellular organelles. Fluorescence intensity was higher in KO cells than in the WT cells. Intense fluorescent signal was localized around the nucleus in KO (b) compared to diffused cytoplasmic staining in WT cells (a). (Data is representative of 8–10 cells for each type and n = 2 independent experiment). Scale bars: 20 μm. Bar graph on the right is the quantification of calcium signal. *, P>0.05 by students t test.

Excessive accumulation of undigested cargo due to lysosomal malfunction is a hallmark feature of Lysosomal Storage Diseases (LSDs) [37]. Changes in calcium level have been shown to be involved in these diseases. Moreover, recently, it was shown that a lysosomal calcium channels play an important role in signaling organelles to degrade its contents. Therefore, we next determined the calcium level in the KO lenses. LECs derived from WT and KO lenses were stained with a Ca^2+^ dye (calcium Orange, Fig 7). Ca^2+^fluorescence intensity was significantly higher in KO-LECs than that in WT-LECs. Moreover, in KO cells, the calcium staining seems to be relatively more intense around the nucleus (Fig 7 panel b), compared to mostly diffused cytoplasmic staining in WT cells (Fig 7 panel a) suggesting that the calcium homeostasis is affected in KO lenses.

### Cytoskeleton Integrity is Impaired in KO Lenses

**Fig 8:**
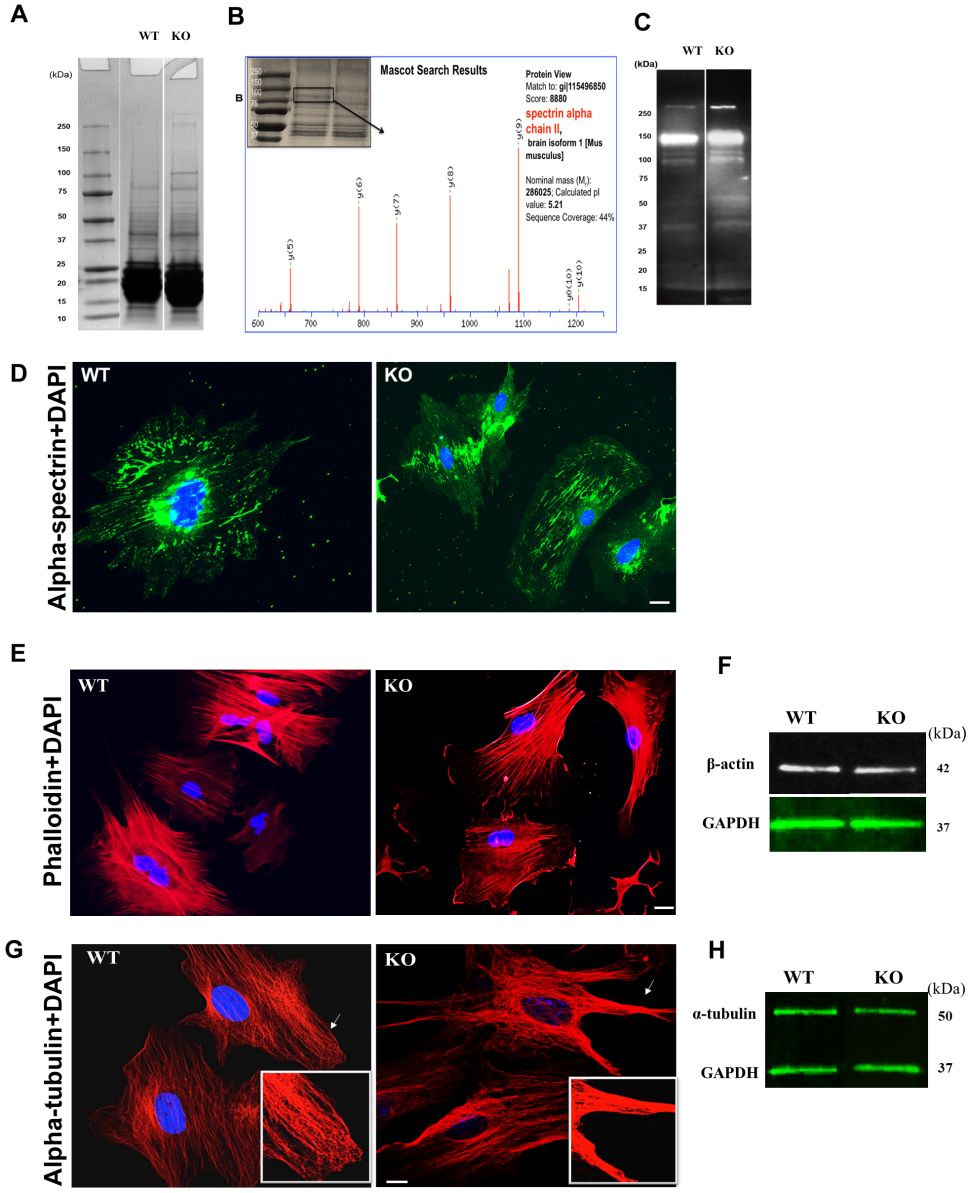
Integrity of cytoskeletal network is affected in KO lenses. Expression of alpha spectrin is altered in KO. **A**. SDS-PAGE showing the protein profile of 1-month old WT and KO lens homogenates. The expression of 120 kDa and 240 kDa protein band was increased in 1-month old KO lenses (shown by an arrow) n = 5 independent experiment. **B**. Identification of 120 kDa protein as a fragment of alpha spectrin by mass spectrometry. **C**. Western blot analysis of 1-month old lens homogenates probed with anti-alpha spectrin antibody showing an increased degradation of alpha spectrin in KO lenses. n = 2 independent experiment. **D**. Expression pattern of alpha-II spectrin in LECs. Immunostaining of LECs with alpha-II spectrin antibody showing the cytoskeletal meshwork of α-spectrin in WT LECs. In contrast, in KO LECs the α-spectrins meshwork are aggregated. Data is representative of 6–8 cells for each type. Scale bars: 20 μm. **E**. LECs were treated with F-actin phalloidin to visualize the actin polymerization. KO LECs showed a reduction in F-actin staining compared to WT LECs. n = 2 independent experiment. Scale bars: 20 μm. **F**. Western blots of 1-month-old lens homogenates probed with a β-actin antibody. Compared to WT the expression of β-actin was not altered in KO lenses. **G**. Immunostaining of LECs with the **α**-tubulin antibody. WT and cells show well-spread tubulin strands compared to KO LECs. In KO cells tubulin strands are aggregated, and the aggregation is prominent near the edges (shown by arrow and inset panel; enlarged area). Data is representative of 6–8 cells for each type Scale bars: 20 μm. **H**. Western blots of 1-month old lens homogenates probed with **α** -tubulin antibody showing an unchanged level of **α**-tubulin protein in KO lenses.

During SDS-PAGE analysis of 1-month old lens homogenates, an increased expression of a protein of ~120 kDa was observed in KO lenses (Fig 8A). Further mass spectrometric analysis identified the 120 kDa band as an α-spectrin fragment. (Fig 8B) Alpha II-spectrin is a 240 kDa cytoskeletal protein that cross-links actin to the cell membrane, and is required for maintaining the stability, structure, and shape of the cell membranes. Western blot analysis using an antibody specific to α-spectrin showed an increased expression and degradation of full length α-spectrin (240 kDa) in KO lenses (Fig 8C). To further evaluate the expression pattern of the alpha spectrin, cultured LECs were immunostained for α-spectrin. In WT cells, immunostaining showed a nicely cross-linked meshwork of α-spectrin in the entire cell with intense staining around the nucleus. In contrast, in KO LECs, spectrin seemed to be partioned and aggregated (Fig 8D). Since spectrin is involved in the cross-linking of β-actin to the cell membrane, we next determined the expression pattern of β-actin in KO lenses. Western blot analysis with anti-β-actin antibody showed no major difference in β-actin protein levels in WT and KO lenses (Fig 8F). However, staining LECs with phalloidin F-actin showed relatively reduced staining in KO LECs compared to WT cells, suggesting that actin polymerization is affected in KO lenses. Next, we also determined the expression pattern of α-tubulin in KO lenses. Similar to β-actin, western blot results showed that the amount of α–tubulin protein was unchanged in the lenses of KO, compared to its levels of α–tubulin protein in WT lenses (Fig 8H). However, when cultured LECs were stained with anti-α–tubulin antibody, in KO LECs, the tubulin strands were aggregated and clumped together and aggregated, and the aggregation was found to be more intense around the edges of the cells (enlarged area), relative to the smooth and even staining in WT LECs (Fig 8G).

### Calpain-3 is activated in KO Lenses

The increased expression and extensive fragmentation of alpha II spectrin and the aggregation of tubulin suggested an accelerated proteolysis of cytoskeletal proteins in KO lenses. Since alpha II spectrin is a known substrate of calpain [38, 39], the increased degradation of spectrin suggests that the enzyme activity is likely to be elevated in KO lenses.

**Fig 9:**
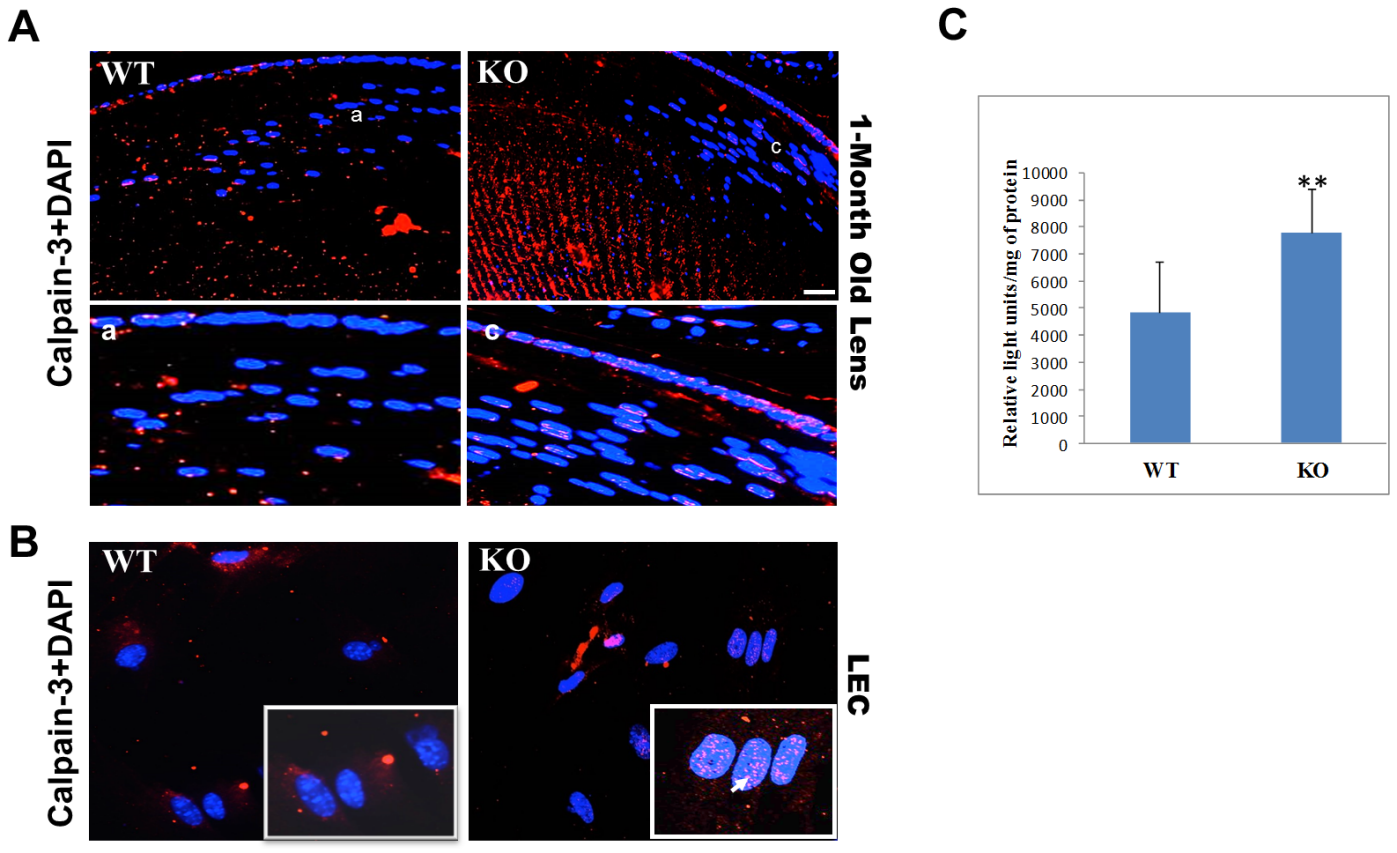
Calpain-3 is activated in KO lenses. **A**. Immunostaining of 1-month old lens sections with anti-calpain Lp-85 antibody. KO lens show an increased expression of calpain compared to WT. Inset images a and b are enlarged areas of the equatorial region of WT and KO lens sections respectively, demonstrating positive calpain staining in the nuclei in KO compared to WT lenses. **B**. Cultured LECs immunostained with anti-calpain Lp-85 antibody. KO cells clearly show calpain staining on the nucleus, compared to WT cells that show calpain staining around the nucleus. Inset images are enlarged areas to visualize the nuclear staining. Data is representative of 6–8 cells for each type. Scale bars: A&B: 50 um and a, b, c and inset images: 20 um. **C**. Determination of calpain activity using Suc-LLVY-AMC calpain substrate. Calpain activity was plotted as relative light units/mg of protein. **, P>0.01 students t test.

To further investigate whether the calpain is activated in KO lenses, the expression of calapin-3 was detected in 1-month old lens sections and in cultured lens epithelial cells. The outer cortical fiber cells showed 3-fold increase in calpain-3 expression in KO lenses compared to its expression in the corresponding regions in WT lenses (Fig 9A). Strikingly, in KO lenses, 60–70% of cortical fiber cell nuclei showed positive calpain-3 staining (Fig 9A, panel c). Consistent with this observation the cultured KO lens epithelial cells also showed clear calapin-3 immunostaining in the nuclei. Additionally, calapin-3 staining in KO cells was much more scattered in the cytosol compared to the localized staining around the nuclei in WT cells (Fig 9B). These observations were further strengthened by the calpain activity assay results carried out using luminescent Suc-LLVY-AMC calpain substrate, where KO lens homogenates showed 60% increase in calpain activity compared to WT (Fig 9C).

## Discussion

In this study we have generated a complete KO mouse model, which carries a germ line deletion of the CRYβA3/A1 gene. For the first time we have shown that the targeted deletion of CRYβA3/A1 gene in mouse leads to development of congenital nuclear cataract and persistence of fetal vasculature (PVF), and causes several cellular defects, including defective clearance of nuclear, mitochondrial and autophagic cargo, altered calcium homeostasis, calpain activation and cytoskeletal defects.

The association between lack of βA3-crystallin function and the PFV condition was also previously observed in the Nuc1 (βA3-crystallin mutant) rat [40]. Studies on the mutant rat suggested that βA3-crystallin is required for astrocyte template formation and that it is essential for the remodeling of retinal vessels. Our βA3-crystallin KO mice showing PFV condition further supports the role of βA3-crystallin in retinal vessel remodeling. Future studies should be able to provide greater insight about the exact mechanism by which βA3-crystallin regulates the developmentally programmed regression of fetal hyaloid vasculure.

With respect to the cellular defects, our data demonstrate that cargo clearance is impaired in KO lenses, as evidenced by the retention of degraded nuclear debris in the cortical and nuclear regions of the lens, the presence of autophagic cargos suggested by LC3-II and p62/SQSTM1 and poly-ubiquitin staining in the lens epithelium and in outer cortical fiber cells, the presence of damaged mitochondria in the lens epithelium and the presence of cytoplasmic debris in the nuclear region of the KO lenses. Further, the results from co-localization of LC3B/MAP1LC3B with lysosomes suggested that autophagic cargo accumulates in KO lenses due to dysfunctional lysosomes. Autophagy is important for the maintenance of lens homeostasis through the degradation of damaged proteins and mitochondria as well as through the clearance of mitochondria during fiber cell maturation to form the OFZ of the lens [41]. Failure to degrade and recycle the accumulated cargo and damaged mitochondria would affect the energy status of the lens. ATP energy is required for the functioning of several enzyme and transport systems, including fiber cell elongation and cytoskeletal remodeling. Therefore, a block in autophagy due to defective lysosomes in KO lenses could have an additional effect on other energy-driven processes.

Our observation that lack of βA3-crystallin leads to block in autophagy is consistent with earlier reports of loss of βA3-crystallin function in RPE and astrocytes. It is reasonable to believe that, similar to RPE and astrocytes, βA3-crystallin is also required for maintaining the pH of the lysosome in the lens. Thus, an increase in lysosomal pH in the absence of βA3-crystallin is responsible for the accumulation of undigested cargos in KO lenses. However, in contrast to RPE and astrocytes in which βA3-crystallin is a lysosomal resident protein, in the lens βA3-crystallin is expressed abundantly in the cytosol. Moreover, βA3-crystallin has a calcium-binding domain and has been shown to bind calcium with moderate affinity, collectively suggesting that βA3-crystallin likely plays additional roles in the lens in contrast to the retinal tissues. Indeed, the KO cells that lack the expression of βA3-crystallin showed increased calcium level compared to WT cells, further suggest its role in regulation of calcium homeostasis in the lens. Interestingly, the importance of calcium channels in LSDs has been unraveling in recent studies. For example, lack of mucolipin (Trpml1)-calcium channel protein in mice leads to the development of LSDs [42] and in FIG4 (PI3,5P2-specific phosphatase deficient mice, release of intralysosomal calcium by activation Ca^2+^ channel TPC2 improves the lysosomal storage phenotype [43]. The extensive accumulation of calcium dye in KO cells around the nucleus implies that the lysosomal Ca^2+^ efflux must be inhibited in KO cells. We speculate that the increased cytoplasmic calcium in the absence of βA3-crystallin may suppress lysosomal Ca^2+^ efflux and cause accumulation of cargo in lysosomes. Additional studies are required to prove that lysosomal Ca^2+^ efflux is suppressed in βA3-crystallin KO lenses.

The increased expression and degradation of alpha II spectrin, the increased aggregation of the cyotoskeleton, the increased partitioning of lens proteins into membrane fractions (data not shown) all implicated that calpain is activated in KO lenses. Moreover, our preliminary RNA seq data showed that calpain-3 mRNA is up regulated in KO lenses relative to the control lenses. In addition, the increased expression and translocation of calpain-3 protein in KO lenses further supported that calpain-3 is activated in KO lenses. Indeed, calpain activity assay using luminescent calpain substrate Suc-LLVY- AMC, showed 60% increased calpain activity in KO lenses compared to WT lenses. Together, these data strongly provide evidence that the calcium homeostasis is altered and calpain is activated in KO lenses.

Beta-crystallins have been shown to bind calcium with moderate affinity, and earlier studies have indicated linkage between βB2-expression in non-lenticular tissue and calcium-dependent stress management [44]. Therefore, it is reasonable to believe that in the absence of βA3-crystallin, calcium homeostasis is imbalanced, and affects the calcium mediated proceses in KO lenses. However, exactly how βA3-crystallin regulates Ca^2+^ homeostasis in lens needs to be studied further. It has been shown that lysosome-calcium signaling controls the activities of calcinurin phosphatase (Ca^2+^ and calmodulin-dependent serine/threonine protein phosphatase 2B) [45] and its substrate, bHLH-leucine zipper transcription factor EB (TFEB), which belongs to the microopthalmia-transcription factor E family, and is a master regulator of lysosome biogenesis and autophagy [46–48]. It binds to 10 bp palindromic sequence called CLEAR element, which is found in many lysosomal genes including cathepsins, calcium channel TRPMl1 and lysosomal membrane protein [47, 49]. Interestingly, TFEB also links mTOR to V-ATPase expression and endocytosis [50]. Therefore, it would be important to know in future studies whether the TFEB activity is affected, and which lysosomal gene expressions are altered in KO lenses.

Since in the present study we used a complete KO model which lacks the expression of βA3-crystallin in both lens and retinal tissues that would complicate the understanding of βA3-crystallin function in the lens. We have also recently generated a lens-specific (Mlr-10) conditional KO of βA3-crystallin. Similar to complete KO, conditional KO mice also develop congenital nuclear cataracts (unpublished data). Currently, studies are underway using the conditional KO of βA3-crystallin to determine how βA3-crystallin regulates the calcium homeostasis in the lens.

## Summary

We have generated CRYβA3-crystallin KO mice that develop congenital nuclear cataract. The result of phenotypic characteristics of βA3-crystallin KO mice developing congenital nuclear cataracts is consistent with reported findings. Our results thus confirm an indispensable role of βA3-crystallin in the lens development. Overall, the findings presented here suggest that several factors, including the retention of the hyaloid artery, impaired cargo clearance, and increased protein degradation and aggregation, contribute to the development of nuclear cataract in KO lenses. Functionally, βA3-crystallin is required for maintaining the cytoplasmic calcium levels. The altered cytoplasmic calcium level in the absence of βA3-crystallin leads to, i) inhibition of lysosomal clearance which in turn causes the cargo accumulation, ii) activates the calpain that causes aggregation and degradation of cytoskeletal proteins. These events are responsible for the development of nuclear cataract in βA3- KO lenses. This is the first such study which links the function of βA3-crystallin to calcium imbalance and lysosomal defects. Further studies on the role of βA3-crystallin on calcium homeostasis in the lens will provide more insight into the pathogenesis of congenital cataract.

## Supporting Information

S1 Fig Orientation of lens fiber cells. **A**. Paraffin sections of 1-month-old WT and KO lenses visualized under light microscope. The panel a and b are the central enlarged area of WT and KO lenses respectively. **B**. The lens sections of WT (c) and KO (d) were stained with WGA-TRITC (red) and DAPI (blue). n = 3. Scale bars: 50 μm.
(TIF)

S2 Fig Immunohistochemical staining of 1-month-old lens sections with the cox–iv antibody. (cox-iv: red, DAPI nuclear stain: blue). Intensity of cox-iv staining was increased compared to WT and HET lenses. The OFZ of KO lenses (panel c) showed positive cox-iv staining compared to the corresponding region in WT (panel a) and HET (panel b) lenses. n = 3 lenses. Scale bars:50 μm.
(TIF)

## References

[1] Delaye M, Tardieu A. Short-range order of crystallin proteins accounts for eye lens transparency. Nature. 1983;302(5907):415–7. Epub 1983/03/06. . 6835373 10.1038/302415a0

[2] Clayton RM, Jeanny JC, Bower DJ, Errington LH. The presence of extralenticular crystallins and its relationship with transdifferentiation to lens. Current topics in developmental biology. 1986;20:137–51. . 2420533 10.1016/s0070-2153(08)60660-2

[3] Xi J, Farjo R, Yoshida S, Kern TS, Swaroop A, Andley UP. A comprehensive analysis of the expression of crystallins in mouse retina. Molecular vision. 2003;9:410–9. . 12949468

[4] Horwitz J. Alpha-Crystallin Can Function as a Molecular Chaperone. P Natl Acad Sci USA. 1992;89(21):10449–53. 10.1073/pnas.89.21.10449 ISI:A1992JW79800097. PMC50356 1438232

[5] Blundell T, Lindley P, Miller L, Moss D, Slingsby C, Tickle I, et al The molecular structure and stability of the eye lens: x-ray analysis of gamma-crystallin II. Nature. 1981;289(5800):771–7. . 7464942 10.1038/289771a0

[6] Herbrink P, Van Westreenen H, Bloemendal H. Further studies on the polypeptide chains of beta-crystallin. Experimental eye research. 1975;20(6):541–8. . 1149833 10.1016/0014-4835(75)90221-3

[7] Berbers GA, Hoekman WA, Bloemendal H, de Jong WW, Kleinschmidt T, Braunitzer G. Homology between the primary structures of the major bovine beta-crystallin chains. European journal of biochemistry / FEBS. 1984;139(3):467–79. . 6698025 10.1111/j.1432-1033.1984.tb08029.x

[8] Lampi KJ, Ma Z, Shih M, Shearer TR, Smith JB, Smith DL, et al Sequence analysis of betaA3, betaB3, and betaA4 crystallins completes the identification of the major proteins in young human lens. The Journal of biological chemistry. 1997;272(4):2268–75. . 8999933 10.1074/jbc.272.4.2268

[9] Jobby MK, Sharma Y. Calcium-binding to lens betaB2- and betaA3-crystallins suggests that all beta-crystallins are calcium-binding proteins. The FEBS journal. 2007;274(16):4135–47. 10.1111/j.1742-4658.2007.05941.x . 17651443

[10] Wenk M, Baumgartner R, Holak TA, Huber R, Jaenicke R, Mayr EM. The domains of protein S from Myxococcus xanthus: structure, stability and interactions. Journal of molecular biology. 1999;286(5):1533–45. 10.1006/jmbi.1999.2582 . 10064714

[11] Clout NJ, Kretschmar M, Jaenicke R, Slingsby C. Crystal structure of the calcium-loaded spherulin 3a dimer sheds light on the evolution of the eye lens betagamma-crystallin domain fold. Structure. 2001;9(2):115–24. . 11250196 10.1016/s0969-2126(01)00573-1

[12] Werten PJ, Stege GJ, de Jong WW. The short 5' untranslated region of the betaA3/A1-crystallin mRNA is responsible for leaky ribosomal scanning. Molecular biology reports. 1999;26(3):201–5. . 10532316 10.1023/a:1007046926233

[13] Kannabiran C, Rogan PK, Olmos L, Basti S, Rao GN, Kaiser-Kupfer M, et al Autosomal dominant zonular cataract with sutural opacities is associated with a splice mutation in the betaA3/A1-crystallin gene. Molecular vision. 1998;4:21 . 9788845

[14] Bateman JB, Geyer DD, Flodman P, Johannes M, Sikela J, Walter N, et al A new betaA1-crystallin splice junction mutation in autosomal dominant cataract. Investigative ophthalmology & visual science. 2000;41(11):3278–85. . 11006214

[15] Qi Y, Jia H, Huang S, Lin H, Gu J, Su H, et al A deletion mutation in the betaA1/A3 crystallin gene (CRYBA1/A3) is associated with autosomal dominant congenital nuclear cataract in a Chinese family. Human genetics. 2004;114(2):192–7. 10.1007/s00439-003-1049-7 . 14598164

[16] Ferrini W, Schorderet DF, Othenin-Girard P, Uffer S, Heon E, Munier FL. CRYBA3/A1 gene mutation associated with suture-sparing autosomal dominant congenital nuclear cataract: a novel phenotype. Investigative ophthalmology & visual science. 2004;45(5):1436–41. . 15111599 10.1167/iovs.03-0760

[17] Reddy MA, Bateman OA, Chakarova C, Ferris J, Berry V, Lomas E, et al Characterization of the G91del CRYBA1/3-crystallin protein: a cause of human inherited cataract. Human molecular genetics. 2004;13(9):945–53. 10.1093/hmg/ddh110 . 15016766

[18] Graw J, Jung M, Loster J, Klopp N, Soewarto D, Fella C, et al Mutation in the betaA3/A1-crystallin encoding gene Cryba1 causes a dominant cataract in the mouse. Genomics. 1999;62(1):67–73. . 10585769 10.1006/geno.1999.5974

[19] Srivastava OP, Srivastava K. Characterization of a sodium deoxycholate-activatable proteinase activity associated with betaA3/A1-crystallin of human lenses. Biochimica et biophysica acta. 1999;1434(2):331–46. Epub 1999/10/19. . 10525151 10.1016/s0167-4838(99)00183-1

[20] Gupta R, Chen J, Srivastava OP. A serine-type protease activity of human lens beta A3-crystallin is responsible for its autodegradation. Molecular vision. 2010;16(239–40):2242–52. ISI:000283719500002. 21139689 PMC2994418

[21] Parthasarathy G, Ma B, Zhang C, Gongora C, Samuel Zigler J Jr, Duncan MK, et al Expression of betaA3/A1-crystallin in the developing and adult rat eye. Journal of molecular histology. 2011;42(1):59–69. 10.1007/s10735-010-9307-1 21203897 PMC3840502

[22] Gehlbach P, Hose S, Lei B, Zhang C, Cano M, Arora M, et al Developmental abnormalities in the Nuc1 rat retina: a spontaneous mutation that affects neuronal and vascular remodeling and retinal function. Neuroscience. 2006;137(2):447–61. 10.1016/j.neuroscience.2005.08.084 . 16289888

[23] Sinha D, Klise A, Sergeev Y, Hose S, Bhutto IA, Hackler L Jr, et al betaA3/A1-crystallin in astroglial cells regulates retinal vascular remodeling during development. Molecular and cellular neurosciences. 2008;37(1):85–95. . 17931883 10.1016/j.mcn.2007.08.016 PMC4943342

[24] Zhang C, Asnaghi L, Gongora C, Patek B, Hose S, Ma B, et al A developmental defect in astrocytes inhibits programmed regression of the hyaloid vasculature in the mammalian eye. European journal of cell biology. 2011;90(5):440–8. 10.1016/j.ejcb.2011.01.003 21354650 PMC3480019

[25] Valapala M, Wilson C, Hose S, Bhutto IA, Grebe R, Dong A, et al Lysosomal-mediated waste clearance in retinal pigment epithelial cells is regulated by CRYBA1/betaA3/A1-crystallin via V-ATPase-MTORC1 signaling. Autophagy. 2014;10(3):480–96. 10.4161/auto.27292 24468901 PMC4077886

[26] Valapala M, Hose S, Gongora C, Dong L, Wawrousek EF, Samuel Zigler J Jr, et al Impaired endolysosomal function disrupts Notch signalling in optic nerve astrocytes. Nature communications. 2013;4:1629 10.1038/ncomms2624 23535650 PMC3718029

[27] Bai F, Xi JH, Wawrousek EF, Fleming TP, Andley UP. Hyperproliferation and p53 status of lens epithelial cells derived from alphaB-crystallin knockout mice. The Journal of biological chemistry. 2003;278(38):36876–86. 10.1074/jbc.M304010200 . 12826669

[28] Testa G, Schaft J, van der Hoeven F, Glaser S, Anastassiadis K, Zhang Y, et al A reliable lacZ expression reporter cassette for multipurpose, knockout-first alleles. Genesis. 2004;38(3):151–8. 10.1002/gene.20012 . 15048813

[29] Mizushima N, Yamamoto A, Matsui M, Yoshimori T, Ohsumi Y. In vivo analysis of autophagy in response to nutrient starvation using transgenic mice expressing a fluorescent autophagosome marker. Molecular biology of the cell. 2004;15(3):1101–11. 10.1091/mbc.E03-09-0704 14699058 PMC363084

[30] Matsui M, Yamamoto A, Kuma A, Ohsumi Y, Mizushima N. Organelle degradation during the lens and erythroid differentiation is independent of autophagy. Biochemical and biophysical research communications. 2006;339(2):485–9. 10.1016/j.bbrc.2005.11.044 . 16300732

[31] Morishita H, Eguchi S, Kimura H, Sasaki J, Sakamaki Y, Robinson ML, et al Deletion of autophagy-related 5 (Atg5) and Pik3c3 genes in the lens causes cataract independent of programmed organelle degradation. The Journal of biological chemistry. 2013;288(16):11436–47. 10.1074/jbc.M112.437103 23479732 PMC3630873

[32] Chen J, Ma Z, Jiao X, Fariss R, Kantorow WL, Kantorow M, et al Mutations in FYCO1 cause autosomal-recessive congenital cataracts. American journal of human genetics. 2011;88(6):827–38. 10.1016/j.ajhg.2011.05.008 21636066 PMC3113247

[33] Glick D, Barth S, Macleod KF. Autophagy: cellular and molecular mechanisms. The Journal of pathology. 2010;221(1):3–12. 10.1002/path.2697 20225336 PMC2990190

[34] Pankiv S, Clausen TH, Lamark T, Brech A, Bruun JA, Outzen H, et al p62/SQSTM1 binds directly to Atg8/LC3 to facilitate degradation of ubiquitinated protein aggregates by autophagy. The Journal of biological chemistry. 2007;282(33):24131–45. 10.1074/jbc.M702824200 . 17580304

[35] Bjorkoy G, Lamark T, Brech A, Outzen H, Perander M, Overvatn A, et al p62/SQSTM1 forms protein aggregates degraded by autophagy and has a protective effect on huntingtin-induced cell death. The Journal of cell biology. 2005;171(4):603–14. 10.1083/jcb.200507002 16286508 PMC2171557

[36] Komatsu M, Waguri S, Koike M, Sou YS, Ueno T, Hara T, et al Homeostatic levels of p62 control cytoplasmic inclusion body formation in autophagy-deficient mice. Cell. 2007;131(6):1149–63. 10.1016/j.cell.2007.10.035 . 18083104

[37] Parkinson-Lawrence EJ, Shandala T, Prodoehl M, Plew R, Borlace GN, Brooks DA. Lysosomal storage disease: revealing lysosomal function and physiology. Physiology. 2010;25(2):102–15. 10.1152/physiol.00041.2009 . 20430954

[38] Siman R, Baudry M, Lynch G. Brain fodrin: substrate for calpain I, an endogenous calcium-activated protease. P Natl Acad Sci USA. 1984;81(11):3572–6. 6328521 10.1073/pnas.81.11.3572 PMC345551

[39] Nixon RA. Fodrin degradation by calcium-activated neutral proteinase (CANP) in retinal ganglion cell neurons and optic glia: preferential localization of CANP activities in neurons. The Journal of neuroscience: the official journal of the Society for Neuroscience. 1986;6(5):1264–71. . 3012012 10.1523/JNEUROSCI.06-05-01264.1986 PMC6568570

[40] Zhang C, Gehlbach P, Gongora C, Cano M, Fariss R, Hose S, et al A potential role for beta- and gamma-crystallins in the vascular remodeling of the eye. Developmental dynamics: an official publication of the American Association of Anatomists. 2005;234(1):36–47. 10.1002/dvdy.20494 . 16003775

[41] Youle RJ, Narendra DP. Mechanisms of mitophagy. Nature reviews Molecular cell biology. 2011;12(1):9–14. 10.1038/nrm3028 . 21179058 PMC4780047

[42] Venugopal B, Browning MF, Curcio-Morelli C, Varro A, Michaud N, Nanthakumar N, et al Neurologic, gastric, and opthalmologic pathologies in a murine model of mucolipidosis type IV. American journal of human genetics. 2007;81(5):1070–83. 10.1086/521954 17924347 PMC2265643

[43] Zou J, Hu B, Arpag S, Hamilton A, Vanoye C, Li J. Reactivation of Lysosomal Calcium Efflux Rescues Abnormal Lysosomal Storage in FIG4 Deficient Cells (S8.007). Neurology. 2015;84(14 Supplement). 10.1523/JNEUROSCI.4442-14.2015 PMC4412898 25926456

[44] Dirks RP, Van Genesen ST, KrUse JJ, Jorissen L, Lubsen NH. Extralenticular expression of the rodent betaB2-crystallin gene. Experimental eye research. 1998;66(2):267–9. . 9533853 10.1006/exer.1997.0439

[45] Klee CB, Crouch TH, Krinks MH. Calcineurin: a calcium- and calmodulin-binding protein of the nervous system. P Natl Acad Sci USA. 1979;76(12):6270–3. 293720 10.1073/pnas.76.12.6270 PMC411845

[46] Medina DL, Di Paola S, Peluso I, Armani A, De Stefani D, Venditti R, et al Lysosomal calcium signalling regulates autophagy through calcineurin and TFEB. Nature cell biology. 2015;17(3):288–99. 10.1038/ncb3114 . 25720963 PMC4801004

[47] Sardiello M, Palmieri M, di Ronza A, Medina DL, Valenza M, Gennarino VA, et al A gene network regulating lysosomal biogenesis and function. Science. 2009;325(5939):473–7. 10.1126/science.1174447 . 19556463

[48] Settembre C, Di Malta C, Polito VA, Garcia Arencibia M, Vetrini F, Erdin S, et al TFEB links autophagy to lysosomal biogenesis. Science. 2011;332(6036):1429–33. 10.1126/science.1204592 21617040 PMC3638014

[49] Medina DL, Fraldi A, Bouche V, Annunziata F, Mansueto G, Spampanato C, et al Transcriptional activation of lysosomal exocytosis promotes cellular clearance. Developmental cell. 2011;21(3):421–30. 10.1016/j.devcel.2011.07.016 21889421 PMC3173716

[50] Pena-Llopis S, Vega-Rubin-de-Celis S, Schwartz JC, Wolff NC, Tran TA, Zou L, et al Regulation of TFEB and V-ATPases by mTORC1. The EMBO journal. 2011;30(16):3242–58. 10.1038/emboj.2011.257 21804531 PMC3160667

