# Supplementary figures and images for: CRYβA3/A1-Crystallin Knockout Develops Nuclear Cataract and Causes Impaired Lysosomal Cargo Clearance and Calpain Activation
Source: PLoS One. 2016 Feb 10;11(2):e0149027. doi: 10.1371/journal.pone.0149027 (PMC4749210; doi:10.1371/journal.pone.0149027)

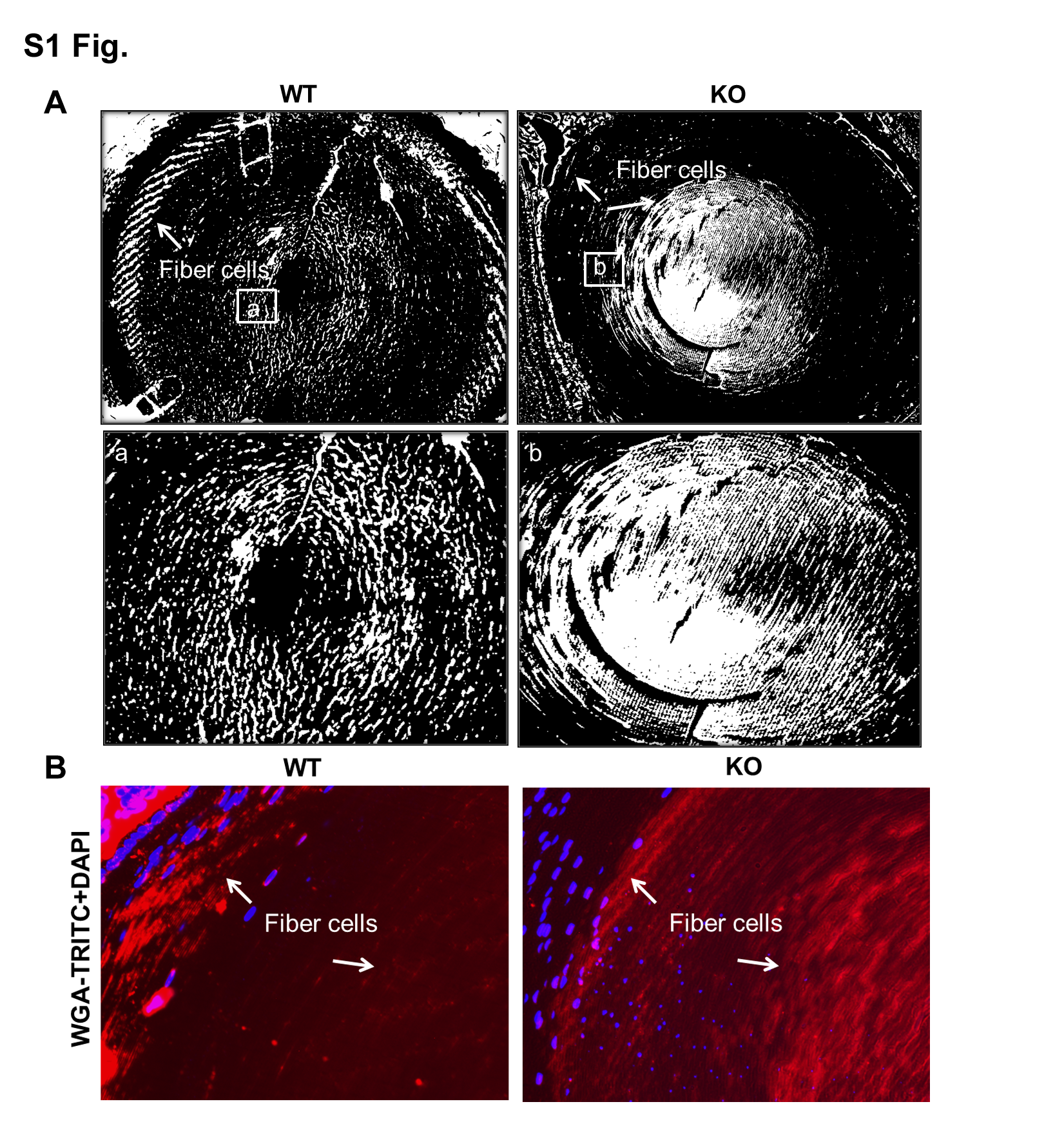

Supplement: S1 Fig — A. Paraffin sections of 1-month-old WT and KO lenses visualized under light microscope. The panel a and b are the central enlarged area of WT and KO lenses respectively. B. The lens sections of WT (c) and KO (d) were stained with WGA-TRITC (red) and DAPI (blue). n = 3. Scale bars: 50 μm. (TIF) [file pone.0149027.s001.tif]

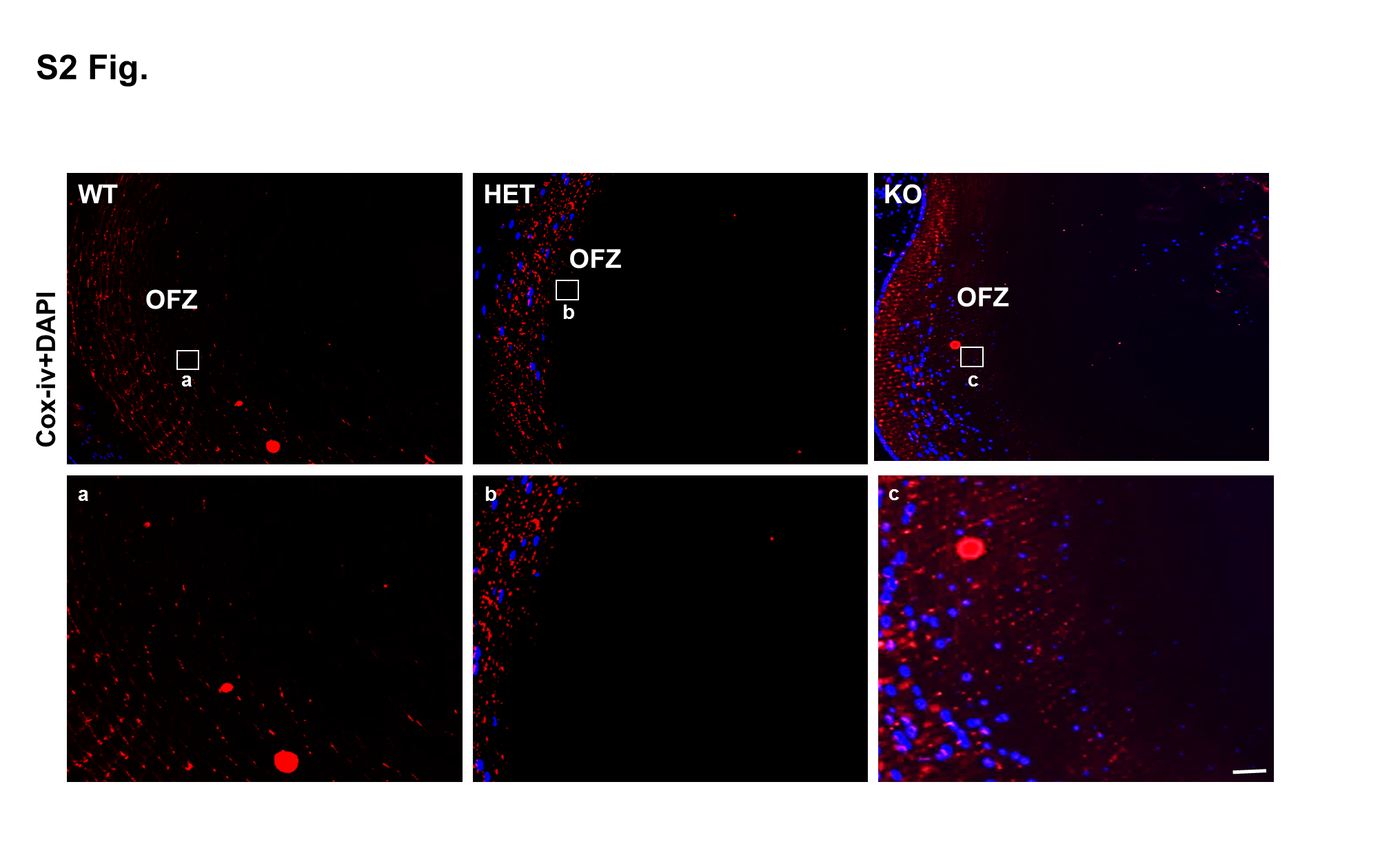

Supplement: S2 Fig — (cox-iv: red, DAPI nuclear stain: blue). Intensity of cox-iv staining was increased compared to WT and HET lenses. The OFZ of KO lenses (panel c) showed positive cox-iv staining compared to the corresponding region in WT (panel a) and HET (panel b) lenses. n = 3 lenses. Scale bars:50 μm. (TIF) [file pone.0149027.s002.tif]
